# Supplementary material for: Contribution of the Tyrosinase (MoTyr) to Melanin Synthesis, Conidiogenesis, Appressorium Development, and Pathogenicity in Magnaporthe oryzae
Source: J Fungi (Basel). 2023 Feb 28;9(3):311. doi: 10.3390/jof9030311 (PMC10059870; doi:10.3390/jof9030311)
Supplement: Supplementary file 1 [file jof-09-00311-s001.zip › Supplementary figures legends.pdf]

Contribution of the Tyrosinase (MoTyr) to melanin synthesis, conidiogenesis, appressorium development, and pathogenicity in *Magnaporthe oryzae*

Xiaoning Fan[1], Penghui Zhang[1], Wajjiha Batool[1], Chang Liu[1], Yan Hu[1], Yi Wei[1], Zhengquan He[2] and Shi-Hong Zhang[1] *

1 The Key Laboratory for Extreme-Environmental Microbiology, College of Plant Protection, Shenyang Agricultural University, Shenyang 110866, China
2 Key Laboratory of Three Gorges Regional Plant Genetics & Germplasm Enhancement (CTGU), Biotechnology Research Center, China Three Gorges University, Yichang 443000, China
* Correspondence: zhangsh89@syau.edu.cn

Supplement Figure S1: Melanin synthesis pathway. A: DHN-melanin synthesis pathway; PKS, polyketide synthase; B: L-DOPA-melanin synthesis pathway. L-DOPA, 3,4-dihydroxyphenylalanine; DQ, Dopaquinone; DHI, 5, 6-dihydroxyindole DHICA; 5,6-dihydroxyindole-2-carboxylic acid; TRP1, tyrosinase-related protein 1; TRP2, tyrosinase-related protein 2.

Supplement Figure S2: Construction of vectors and verification of mutants. (A) Construction of *MoTyr* gene knockout vector. (B) Verification of MoTyr knockout mutant strains. M stands for DNA marker (2000 bp; 1000 bp; 750 bp; 500 bp; 250 bp; 100 bp).

Supplement Figure S3: Growth characteristics of wild-type and Δ*MoTyr* mutant stains. (A) Observation of vegetative growth. (B) Colony diameter statistics. (C) Conidia morphology of wild-type and mutants; Scale bar =20 μm. ns represent not significant; $p<0.05$.

Supplement Figure S4： Conidia germination and appressoria formation. (A) Development of conidia in different time periods. (B) Statistical analysis of conidia germination rate of different strains. (C) The formation of appressoria of different mutant strains at different times. Scale bar =20 μm; ns represent not significant; $p<0.05$.
