# Supplementary figures and images for: Contribution of the Tyrosinase (MoTyr) to Melanin Synthesis, Conidiogenesis, Appressorium Development, and Pathogenicity in Magnaporthe oryzae
Source: J Fungi (Basel). 2023 Feb 28;9(3):311. doi: 10.3390/jof9030311 (PMC10059870; doi:10.3390/jof9030311)

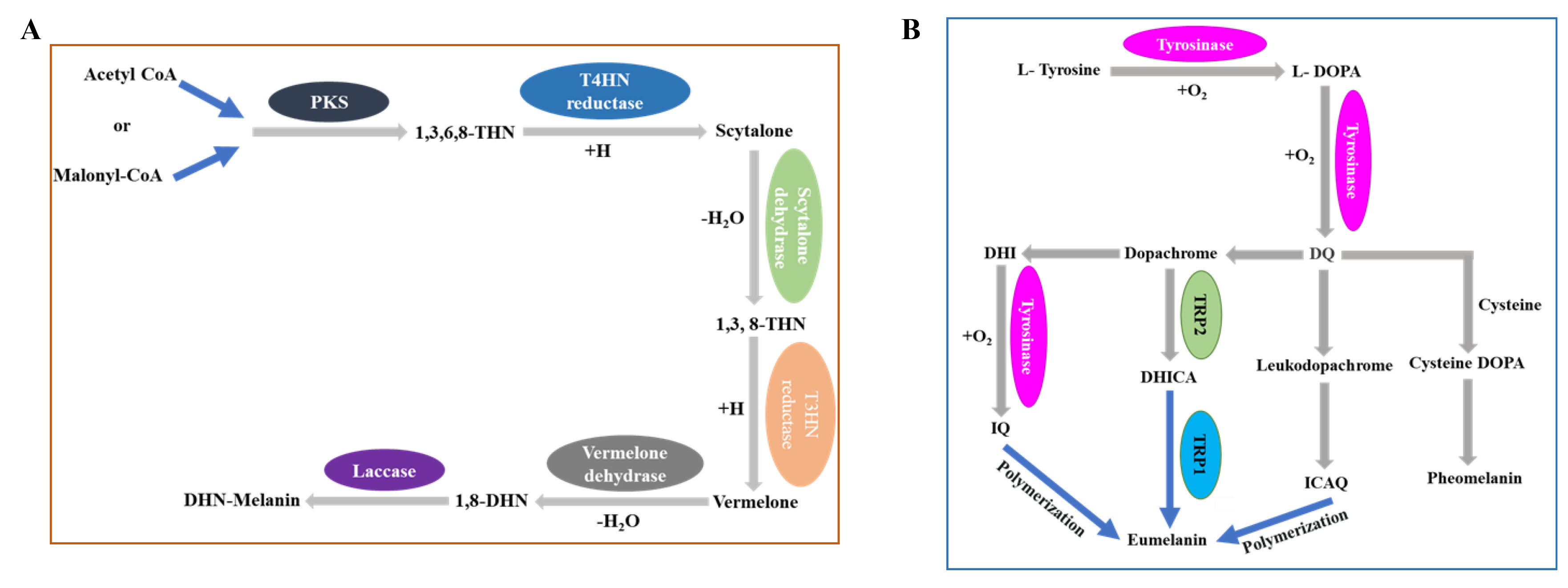

Supplement: Supplementary file 1 [file jof-09-00311-s001.zip › Supplement Figure S1.tif]

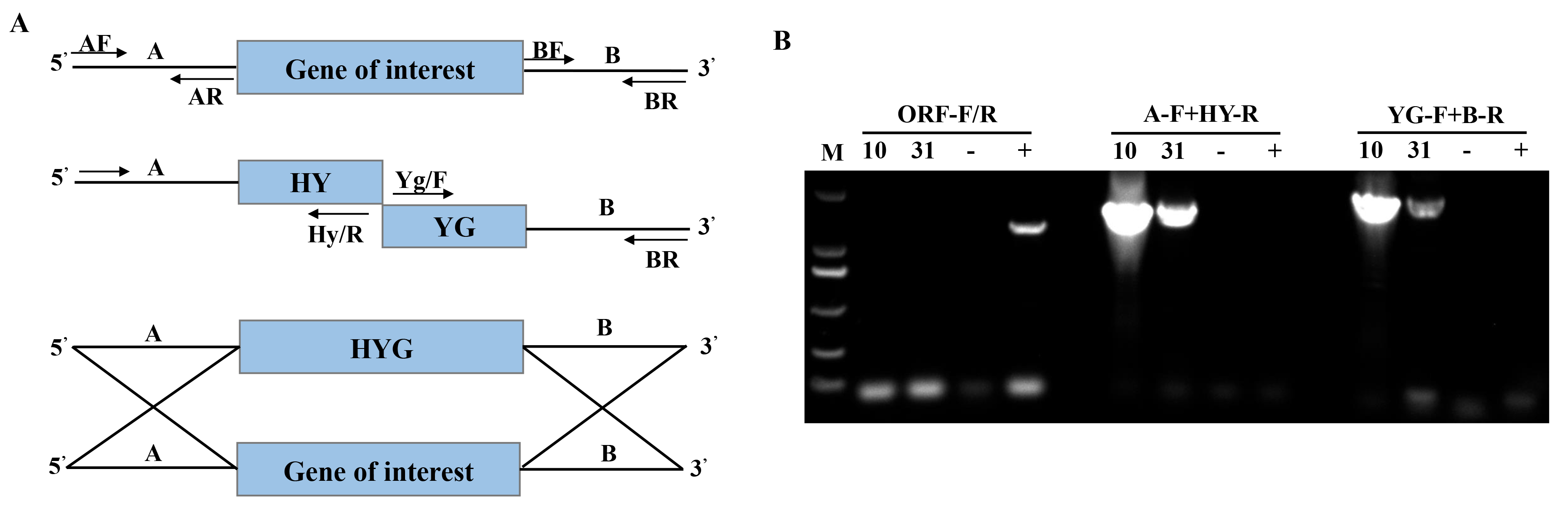

Supplement: Supplementary file 1 [file jof-09-00311-s001.zip › Supplement Figure S2.tif]

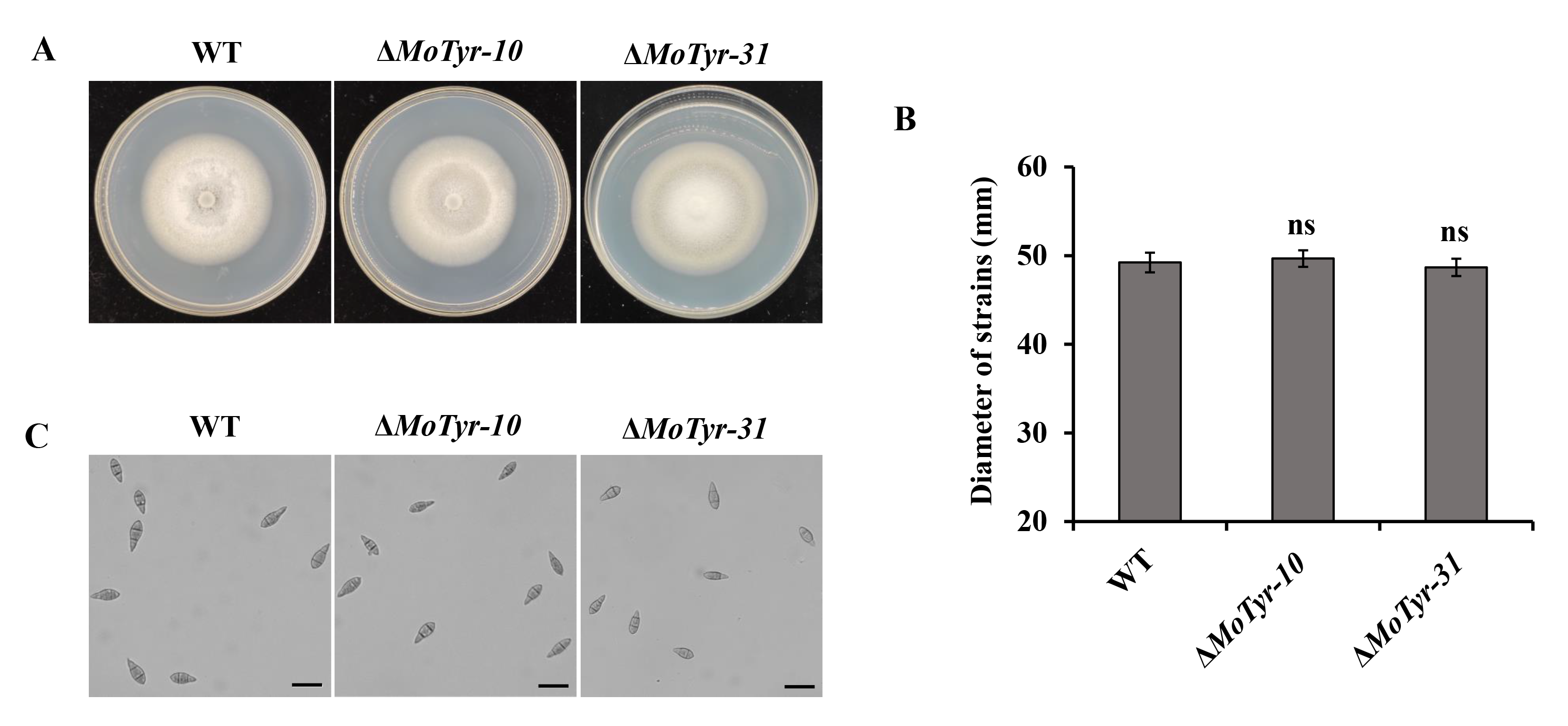

Supplement: Supplementary file 1 [file jof-09-00311-s001.zip › Supplement Figure S3.tif]

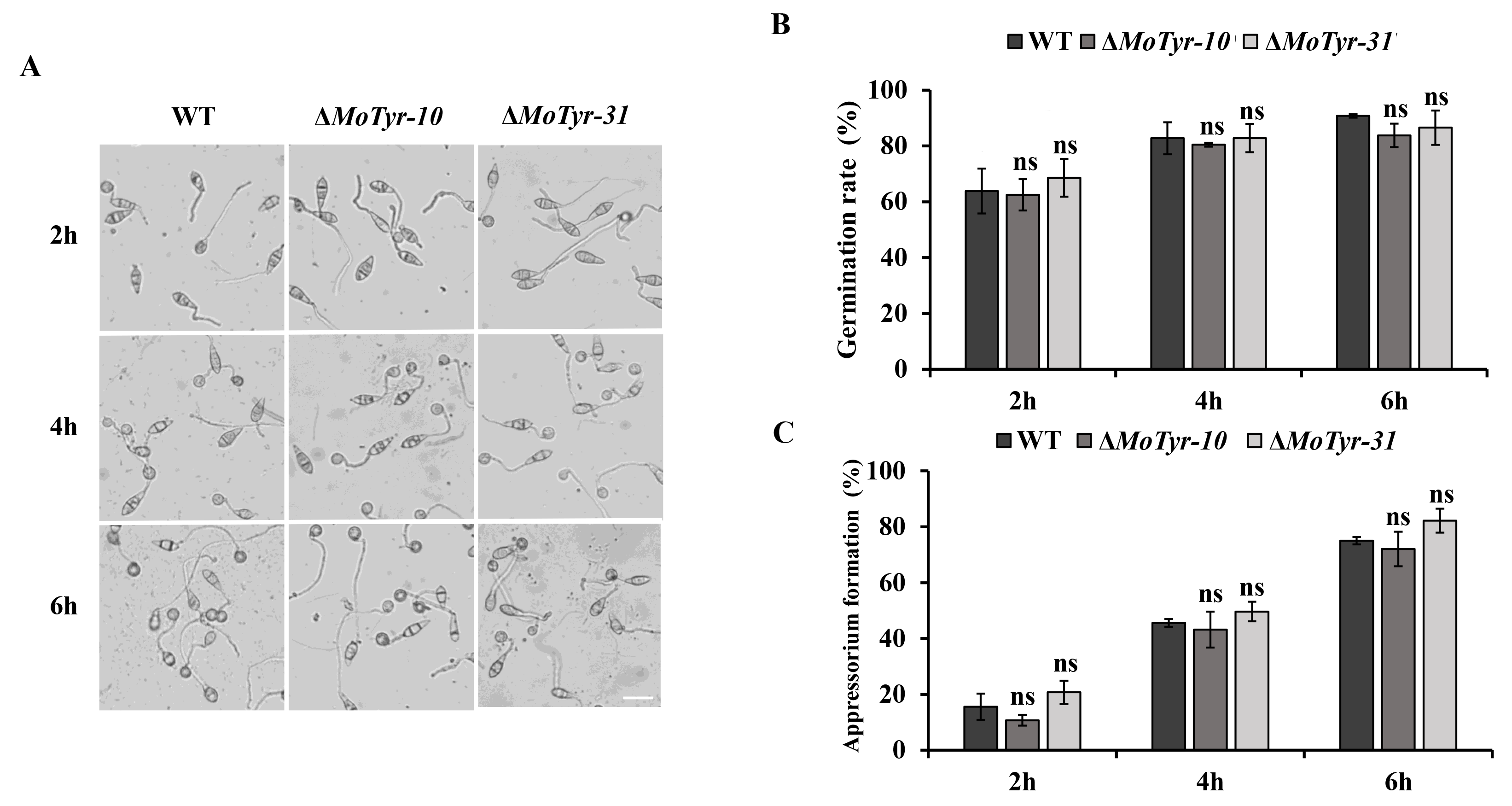

Supplement: Supplementary file 1 [file jof-09-00311-s001.zip › Supplement Figure S4.tif]
